# Semantic and Syntactic Predictions in Reading Aloud: Are Good Predictors Good Statistical Learners?

**DOI:** 10.5334/joc.363

**Published:** 2024-05-09

**Authors:** Elisa Gavard, Johannes C. Ziegler

**Affiliations:** 1 Aix-Marseille Univ, CNRS, Centre de Recherche en Psychologie et Neuroscience (UMR 7077), Marseille, France

**Keywords:** context effect, reading aloud, semantic prediction, syntactic prediction, statistical learning

## Abstract

Recent research suggests that becoming a fluent reader may partially rely on a domain-general statistical learning (SL) mechanism that allows a person to automatically extract predictable patterns from the sensory input. The goal of the present study was to investigate a potential link between SL and the ability to make linguistic predictions. All previous studies investigated quite general levels of reading ability rather than the dynamic process of making linguistic predictions. We thus used a recently developed predictive reading task, which consisted of having participants read aloud words that were preceded by either semantically or syntactically predictive contexts. To measure the componential nature of SL, we used a visual and an auditory SL task (VSL, ASL) and the classic serial reaction time task (SRT). General reading ability was assessed with a reading speed/comprehension test. The study was conducted online on a sample of 120 participants to make it possible to explore interindividual differences. The results showed only weak and sometimes even negative correlations between the various SL measures. ASL correlated positively and predicted general reading ability but neither semantic nor syntactic prediction effects. Similarly, one of the SRT measures was significantly associated with reading level and reading speed but not with linguistic prediction effects. In sum, there is little evidence that domain-general SL is a good predictor of people’s ability to make domain-specific linguistic predictions. In contrast, SL shows a weak but significant association with general reading ability.

## Introduction

An important part of fluent and skilled reading is the ability to predict upcoming words and integrate them into the preceding context (DeLong et al., 2005; Ehrlich & Rayner, 1981; Smith & Levy, 2013). Such predictions can be made on the basis of semantic (Kutas & Federmeier, 2011), syntactic (Ferreira & Clifton, 1986) or pragmatic (Ni et al., 1996) information. Prediction is a rather general mechanism in cognition (Bar, 2009; Lupyan & Clark, 2015) and it is seen as one of the main cognitive mechanisms involved in language comprehension, language production and reading (Chang et al., 2006; Federmeier & Kutas, 1999; Pickering & Garrod, 2013; Staub, 2015). Indeed, listeners constantly predict future utterances as they listen (Johnson et al., 2013), speakers predict their own utterances as they speak (Pickering & Gambi, 2018) and readers predict upcoming words as they read (Staub, 2015). Linguistic predictions are already made in very young children. For instance, when two-year old children hear the sentence “The boy eats a big cake” they tend to fixate edible objects in a visual scene (a cake) right after they hear the semantically constraining verb *eats* and prior to hearing the word *cake* (Mani & Huettig, 2012). One of the most fascinating hypotheses is whether the ability to make predictions in the domain of language relies on the domain-general ability of extracting regularities from a quasi-regular system and apply this statistical knowledge to predict future events, which is referred to as *statistical learning* or SL (Arciuli & Conway, 2018; Saffran et al., 1996; Siegelman, Bogaerts, Christiansen, et al., 2017).

In their seminal work, Saffran et al. (1996) showed that young infants as early as 8 months were able to track statistical patterns in continuous speech and use this information for detecting word boundaries. Subsequent research confirmed that SL ability was associated with different aspects of language acquisition, such as word segmentation (Swingley, 2005; Thiessen et al., 2013), phonological learning (Maye et al., 2002; Spencer et al., 2015; Thiessen & Saffran, 2003, 2007), and syntactic learning (Kidd, 2012; Kidd & Arciuli, 2016; Thompson & Newport, 2007). It has been shown that individual differences in SL tasks predict sentence comprehension in adults (Misyak & Christiansen, 2012). A recent meta-analysis by Ren et al. (2023) that was based on 42 articles with 53 independent samples confirmed a significant relation between SL and language-related outcomes even though the size of that relation was only moderate (r = 0.236).

In recent years, there has been growing interest in investigating the link between SL and reading. Indeed, the initial stages of learning-to-read are all about learning the quasi-regular mapping of letters onto sounds (Plaut et al., 1996; Ziegler et al., 2014), which might well rely on statistical learning (Seidenberg & MacDonald, 2018). In one of the first studies, Arciuli & Simpson (2012) investigated the relationship between SL and reading ability in typically developing children and healthy adults. SL was measured using visually presented stimuli within a triplet learning paradigm and reading ability was assessed with a standardized reading test. SL accounted for a small but significant amount of variance in reading ability, even after controlling for age and attention, suggesting that SL is positively associated with higher reading ability in the general population. Subsequent research showed that individual differences in an auditory but not visual SL were correlated with sentence and nonword reading in both children and adults (Qi et al., 2019).

However, a number of studies found no or extremely weak correlations between SL and reading (Haebig et al., 2017; Siegelman & Frost, 2015). For example, Witteloostuijn et al. (2021) studied the link between literacy skills (reading and spelling) and SL using a Serial Reaction Time (SRT) and a visual SL task and found no evidence for a link between SL and reading. Similarly, Schmalz et al. (2019) used an SRT and an artificial grammar learning (AGL) task to assess SL and found again no significant correlation between these SL measures and word and nonword reading fluency. The picture does not get much clearer when considering studies that investigated SL deficits in developmental dyslexia (DD). Indeed, some studies found that children or adults with DD had a significant SL deficit (Lukasova et al., 2016; Ozernov-Palchik et al., 2023; Przekoracka-Krawczyk et al., 2017), while others did not find an SL deficit in DD (Inácio et al., 2018; Staels & Van Den Broeck, 2017).

Given the mixed findings in the literature concerning the link between SL abilities and cognitive capacities, Siegelman et al. (2017) argued that one should be cautious about drawing firm conclusions from a lack of correlations in a single study for a number of reasons. First, different SL tasks do not seem to measure a general unified capacity because they tend to correlate weakly and produce qualitatively different results across modalities (Frost et al., 2015). Indeed, Arciuli and colleagues called for a refinement of SL theory, which should be thought of as a componential, rather than a domain-general construct (Arciuli, 2018; Arciuli & Conway, 2018; Ren et al., 2023). Admittingly, if SL reflects a componential ability rather than a unified construct, no single task can fully capture the various facets of SL. Second, most studies that were interested in inter-individual differences in SL have employed the original tasks that were designed for measuring group-level differences. As nicely demonstrated by Siegelman et al. (2017), the original tasks, however, tend to be psychometrically weak for assessing inter-individual differences mainly because the number of trials in the test phase is often too small and a large proportion of the sample tends to perform at chance. They argue that “these factors lead to high measurement error, inevitably resulting in low reliability, and thereby doubtful validity” (p. 418). Siegelman et al. (2017), therefore, developed a novel method specifically designed for the measurement of individual differences in visual and auditory SL, which displays substantially superior psychometric properties than previous SL measures.

The goal of the present study was to investigate a potential link between SL and the ability to make linguistic predictions. Indeed, all previous studies investigated quite general levels of reading ability using standardized reading tests (Arciuli & Simpson, 2012; Misyak & Christiansen, 2012) or reading fluency measures (Qi et al., 2019). These are “static” reading performance measures that are not directly linked to the “dynamic” process of making linguistic predictions. We hypothesized that, if anything, SL measures should more strongly predict the ability to make linguistic predictions rather than measures of reading speed or reading fluency.

To measure linguistic predictions, we used our recently developed *predictive reading tas*k, which consisted of having participants read aloud words that were preceded by either semantically or syntactically predictive contexts (Gavard & Ziegler, 2022). We have shown that this task produced strong semantic and syntactic context (priming) effects, that is, reading aloud latencies for the final word in a sequence were faster when it was preceded by a semantically related context (doc – cat – rat – rabbit – mouse) than an unrelated context (shirt – flower – plane – table – mouse). Similarly, reading aloud latencies for the final word were faster when it was preceded by a syntactically predictive context (she – likes – this – little – mouse) as opposed to a syntactically incorrect context (this – likes – little – she – mouse). Note that the target words across all conditions were identical making it possible to measure the effects of making linguistic predictions in a highly controlled fashion. Interestingly, when enough time was given to process the context (~1000 ms per word), syntactic context effects were even larger than semantic context effects despite the fact that syntactic contexts did not make it possible to predict the exact lexical identity of the target word (for discussion see Gavard & Ziegler, 2022).

As concerns the SL tasks, given the concerns about the validity of SL tasks (Siegelman et al., 2017) and the arguments in favor of the componential nature of SL (Arciuli, 2018; Arciuli & Conway, 2018; Ren et al., 2023; Siegelman et al., 2017), we decided to use three SL tasks. First, we used both a visual and an auditory SL task (VSL, ASL), because these segmentation tasks are probably the most commonly used SL measures and they have been found to be related to reading ability (Qi et al., 2019). In particular, we used the novel VSL and ASL tasks developed by Siegelman et al. (2017), in which they managed to increase the sensitivity of measuring interindividual differences in SL. Second, we used a sequential motor learning task, the classic serial reaction time task (SRT, Nissen & Bullemer, 1987), because it allowed us to capture a different, more “dynamic”, measure of implicit (statistical) learning. Implicit learning refers to the acquisition of knowledge without conscious awareness or intention and is thought to rely on the procedural memory system (Berry & Dienes, 1993; Christiansen, 2019; Perruchet & Pacton, 2006; Shanks, 2005). Finally, to measure general reading ability, we added a standardized reading task, in which participants have to read short sentences and must decide as quickly as possible whether they make sense or not (SLS-Berlin, Lüdtke et al., 2019, for a French validation see Grasso et al., 2022). The SLS is an excellent measure of reading ability that taps both reading speed and reading comprehension.

## Method

All material (stimuli), data (except the raw audio recordings due to privacy concerns), and statistical analyses are available on the OSF (https://osf.io/e2msk/).

### Participants

We recruited 170 native French-speaking participants for this study (95 females, 75 males, age = 26.02 years, SD = 5.51 years) through Prolific, a platform for online subject recruitment.^1^ Participants self-reported normal or corrected-to-normal vision, no learning disorders (dyslexia), and no neurological or psychiatric disorders. All participants gave online written informed consent and were paid £9 average reward per hour. Of this sample, 120 participants (61 females, 59 males, age = 26.73 years, SD = 5.86 years) were employed for the primary phase of the study and 50 participants (34 females, 16 males, age = 23.2 years, SD = 3.31 years) in the assessment of the test-retest reliability of the predictive reading tasks and the serial reaction time task.

### Materials

#### Predictive reading task

To assess the effects of semantic prediction, participants had to read aloud target words preceded by a context of semantically related nouns (cat – dog – rabbit – mouse) compared to a sequence of unrelated nouns (table – green – flower – mouse). To assess the effects of syntactic prediction, they had to read aloud the same target words preceded by a context of syntactically correct sentences (she – likes – this – mouse) compared to syntactically incorrect sentences (this – likes – she – mouse). The design and materials were identical to Experiment 3 of Gavard & Ziegler (2022), except that the current study was run online.

For the semantic prediction task, we used 80 target words that were preceded by semantically related or unrelated contexts. The semantic relatedness between all context words was calculated using a distributional vector space model of semantic associations trained on a very large number of words (Frwiki, 11GB, 914,601,321 tokens, DISCO, Kolb, 2008). This allowed us to select from these semantic contexts 80 target words that were the most predictive in a given related context (average cosine value = .75). Note that vector space models compute semantic similarities rather than cooccurrences or associative relationships (for a discussion, see Günther et al., 2019). For the same 80 target words, we created 80 unrelated contexts matched in word length and frequency that had no semantic relatedness according to DISCO (average cosine value = .12).

For the syntactic condition, we constructed 80 sentences, which had the same target words as in the semantic condition. The syntactic contexts were constructed such that the target word was strongly predicted on a syntactic basis but not at all on a semantic basis (verified using the same vector space model as above). Syntactic predictability was calculated using the Universal Dependencies corpus (de Marneffe et al., 2021). For each target word, we calculated conditional probabilities of predicting the grammatical class of the target word taking into account the preceding context (P(target/context) > 0.60). Eighty syntactically nonpredictive contexts were created by using the same target words but scrambling the words of the context such that syntactic prediction was close to zero (P(target/context) < 0.01).

**Figure 1:**
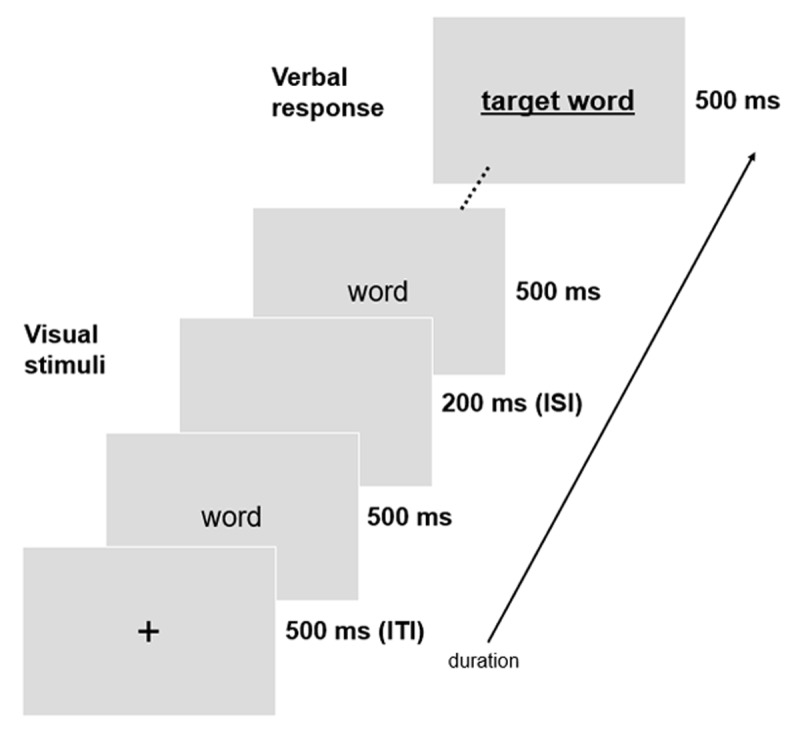
Experimental design and timing of the predictive reading task.

Each trial began with the presentation of a fixation cross in the center of the screen for 500 ms (intertrial interval, ITI). Context words were presented one by one in the center of the screen for 500 ms with a 200 ms interstimulus interval (ISI), in 24pt Lato using black font (or read for the target words) in a white background (see Figure 1). Participants were instructed to read silently (context words) and read aloud words indicated in red (target words) as quickly as possible. Reponses were recorded using the microphone of the participants’ computer. Participants received instructions visually at the beginning of each task.

Individual prediction scores in the predictive reading task were calculated by dividing for all participants the difference between their speed on scrambled minus related trials by their overall response speed (for distributional analyses, see Appendix, Table S1). Test-retest reliability for this novel task was obtained from an independent sample of 50 participants (34 females, 16 males, age = 23.2 years, SD = 3.31 years) who performed the predictive reading task twice separated by a minimum of one day and no more than one week (mean time between sessions = 2.98 days, SD = 2.14 days) between the two sessions. We examined cross-session correlations using Pearson correlation on our individual prediction scores and we found a significant test-retest reliability for the predictive reading task of *r* = 0.56 (*p* < 0.001, FDR corrected).

#### Serial Reaction Time task

To investigate interindividual differences in SL, we used a classic version of the serial reaction time task (SRT, Nissen & Bullemer, 1987). SRT is a sequential motor learning task, in which participants are usually presented with visual stimuli at different locations on a screen and are instructed to press a button corresponding to the location of the stimulus. In some blocks, the order of stimulus locations follows a sequential pattern, in others, stimulus locations are presented randomly.

For this task, we created a fixed second order conditional (SOC) sequence, ideal for studying implicit sequence learning (Schwarb & Schumacher, 2012). The repetition of this sequence constitutes the sequential condition presented during six blocks of 12 sequences of eight positions. During the 7^th^ block, 96 random positions are presented. A final block (the 8^th^) is added at the end of this task, again with 12 fixed sequences of the sequential condition. Participants were not informed about the repetition of the eight consecutive positions of the sequential condition.

**Figure 2:**
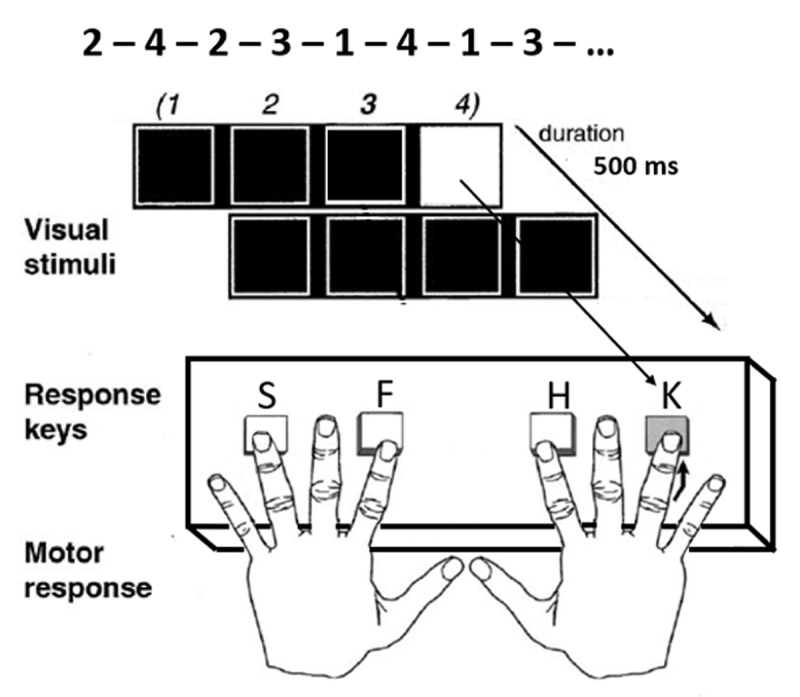
Experimental design and timing of the SRT task (adapted from Schendan et al. (2003)).

Each trial began with four horizontal spatial black squares on a light grey background presented in the center of a monitor screen for 500 ms. Each position was displayed for 500 ms (with a 200 ms inter-stimuli interval) and was indicated by a color change of the square (one of the black squares lights up in white indicating one of the 4 positions of the sequence). Participants were instructed to press a key on a keyboard corresponding to the position of appearance of the white square as quickly and as accurately as possible (press the S key for the 1^st^ position, the F key for the 2^nd^ one, the H one for the 3^rd^ one, and the K key for the 4^th^ position) (see Figure 2).

**Figure S1:**
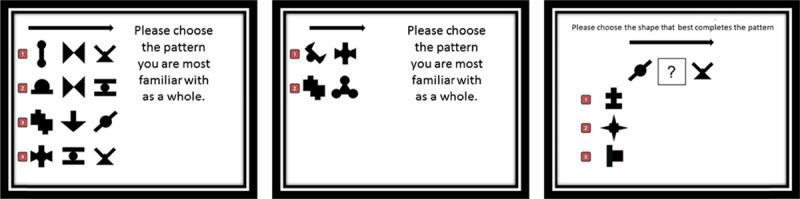
Example of three trials: two “pattern recognition” trials and 1 “pattern completion” trial (taken from Siegelman, Bogaerts, & Frost, 2017). The instructions were presented in French.

Individual SL scores in the SRT task were computed in different ways (Schmalz et al., 2019). Two variables measured the change of performance across repeated blocks: (a) “SRT12” subtracts the mean RT of the 2^nd^ sequential block from the mean RT of the 1^st^ sequential block and (b) “SRT16” subtracts the mean RT of the 6^th^ sequential block from the mean RT of the 1^st^ sequential block. Three variables measured the change of performance between repeated and random blocks: (c) “SRT67” subtracts the mean RT of the 6^th^ sequential block from the mean RT of the 7^th^ random block, (d) “SRT78” subtracts the mean RT of the 8^th^ sequential block from the mean RT of the 7^th^ random block, and (e) “SRT” subtracts the average of the two sequential blocks that preceded and followed the random block (i.e., block 6 and 8) from the mean RT of the 7^th^ random block (Witteloostuijn et al., 2021). The psychometric properties and distributions for all variables are found in Figure S1 of the Appendix. As for the predictive reading scores, we calculated ratio scores by dividing the difference between the participants’ speed on scrambled minus related trials by their overall response speed (Oliveira et al., 2023).

The test-retest reliability of our SRT task was obtained on a new sample of 50 participants (34 females, 16 males, age = 23.2 years, SD = 3.31 years) who did the SRT task twice with a minimum of one day and no more than one week between sessions (mean time between sessions = 2.98 days, SD = 2.14 days. We examined cross-session correlations using Pearson correlation on the individual prediction scores and we found a significant test-retest reliability of *r* = 0.62 (p < 0.001, FDR corrected).

#### Auditory Statistical Learning task

For the auditory modality, we used the exact same task as Siegelman et al. (2018), in which they generated nonlinguistic auditory stimuli that do not implicate prior knowledge about the transitions of phonemes and syllables (as in the typical auditory SL task of Saffran et al., 1996). Sixteen familiar sounds formed the basic elements in the stream (e.g., glass breaking, dog barking, clock ticking, etc).^2^ The 16 sounds were organized into eight triplets: four with transitional probabilities (TPs) of 0.33 and four with TPs of 1. The eight triplets appeared 24 times, one after the other in a random order to create a 10-min familiarization stream, in which participants were instructed to listen to the stream without doing anything. The test-phase consisted of 42 items with a block of 34 pattern recognition items and a block of eight pattern completion items. In each test trial, all options appeared together on the screen (with the internal positions of the target and foils randomized for each item) and participants were asked to select the correct answer using the keyboard (see Appendix, Figure S1). For the full details regarding the construction of foils and test trials, see Table 3 in Siegelman, Bogaerts, & Frost (2017).

#### Visual Statistical Learning task

**Figure S2:**
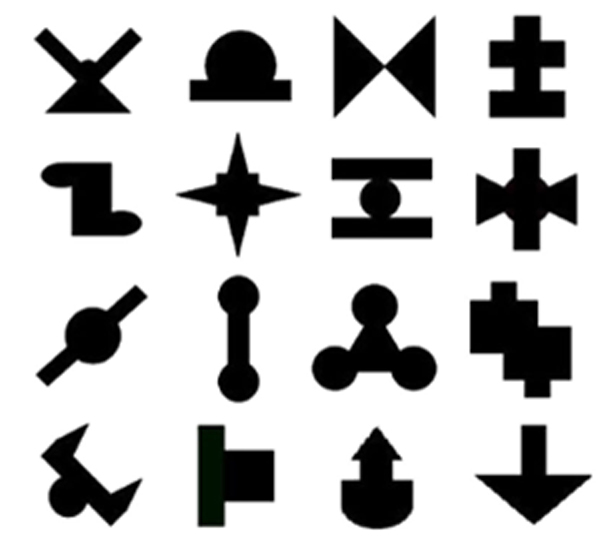
Shapes used in the VSL task (taken from Fiser & Aslin, 2001).

For the visual modality, we used the exact same new visual SL task of Siegelman, Bogaerts and Frost (2017) including 16 complex visual shapes (taken from Fiser & Aslin, 2001, see Appendix, Figure S2). The 16 shapes were organized into eight triplets: 4 with TPs of 0.33 and 4 with TPs of 1. As in the ASL task, the task consisted of a first 10-min familiarization phase, in which the 8 triplets previously organized appeared 24 times randomly. Each shape appeared on the screen for 800 ms, with a 200-ms break between shapes. The test phase was identical to the ASL task with 34 pattern recognition items and 8 pattern completion items. The total score of the ASL and VSL tasks ranged from 0 to 42, based on the number of correct responses in the test.

#### Reading level task

To measure interindividual differences in reading fluency and reading comprehension, we translated the SLS-Berlin (Lüdtke et al., 2019), which is an adult version of the *Salzburger Lese Screening* (SLS) test (Wimmer & Mayringer, 2014). In this task, participants were presented with short sentences, and they were asked to make plausibility judgments as quickly and as accurately as possible after reading each of the sentences (e.g., “The cello is an animal belonging to the category of birds”). Each sentence was shown individually at the center of the screen in 24pt Lato using black font on white background. Participants were asked to press the S key if the sentence was incorrect or the L key if it was correct). Individual reading level scores were derived from the number of correctly judged sentences within a 3-min time frame. The 3-min cut-off was not mentioned.

### Online testing procedure

The study was run online using LabVanced, a unified JavaScript framework for online studies (Finger et al., 2017).^3^ Participants ran the experiment using a web browser and they began by reading through the consent form and general experiment instructions. Demographic variables for participants were collected, such as age and gender, but all the inclusion criterion (native language, learning disorders) were controlled using Prolific before beginning the experiment. A session typically lasted for about one hour.

### Statistical analyses

#### Predictive reading task

The reading aloud responses were recorded via Prolific (wav files). We used CheckVocal (Protopapas, 2007) to determine the onset of each reading aloud response (RT) and judged whether the word had been pronounced correctly. Out of all 30,400 observations, 254 were removed because of missing data. We then removed extreme values (i.e., RTs below 150 ms or above 1500 ms) and we further considered as outliers data points that were above or below 2.5 SD from each individual participants’ mean RT. Outliers were replaced by the cutoff RT corresponding to each participant’s ± 2.5 SD (see Hoaglin et al., 1986). For the RT analysis, we used R (Version 4.3.1.) and *lme4* package (Bates et al., 2015) to perform a linear mixed-effects (LME) analysis with participants as random effects and condition and context as fixed effects. We report unstandardized regression coefficients (b), standard errors (SEs), and t-values. Fixed effects were considered significant if |t| was greater than 1.96 (Baayen, 2008). Fixed effects, random effects, and random slopes were only included if they significantly improved the model’s fit in a forward stepwise model selection procedure.

#### SRT task

We used R to perform two LME analyses, first with blocks as the independent variable and accuracy as the dependent variable, and then with reaction time (RT) of correct responses as the dependent variable. Out of all 92,160 observations (RTs), 7134 were removed because of incorrect responses. We report unstandardized regression coefficients (b), standard errors (SEs), and t-values, considered significant if |t| was greater than 1.96 (Baayen, 2008).

#### Correlations

Because we were interested in the relation between SL, linguistic prediction and reading ability, we conducted a series of correlation analyses using R and the *Hmisc* and *PerformanceAnalytics* packages.

## Results

### Predictive reading task

**Figure 3:**
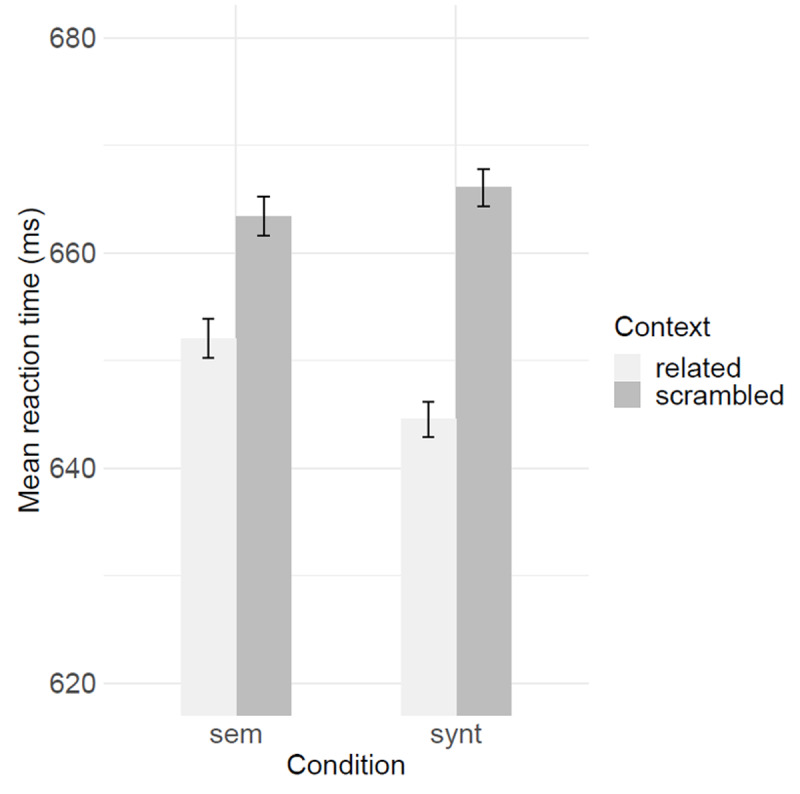
Performance in the predictive reading task. Mean reaction times (ms) are presented as a function of context (related vs. scrambled) and condition (semantic vs. syntactic). Errors bars indicate within-participant standard errors.

Because the RT distribution was slightly skewed, we log-transformed the RT. This resulted in a perfectly normal RT distribution (Skewness: .13, Kurtosis: –0.03; Blanca et al., 2013). For the LME analysis, the final model included condition, context, and the interaction between these variables. As random effects, we included by-participant intercepts, random slopes for condition and context by participants. The results showed a significant effect of context (*b* = 0.008, *SE* = 0.001, *t* = 7.07), no effect of condition but a significant effect of the interaction between the effects of context and condition (*b* = 0.006, *SE* = 0.002, *t* = 3.31). Contrast analysis confirmed that participants were faster at reading the target word following related than unrelated contexts in both conditions with a larger effect of the syntactic condition (*b* = –0.01, *SE* = 0.001, *t* = –10.38; see Figure 3).

### SRT task

**Figure 4:**
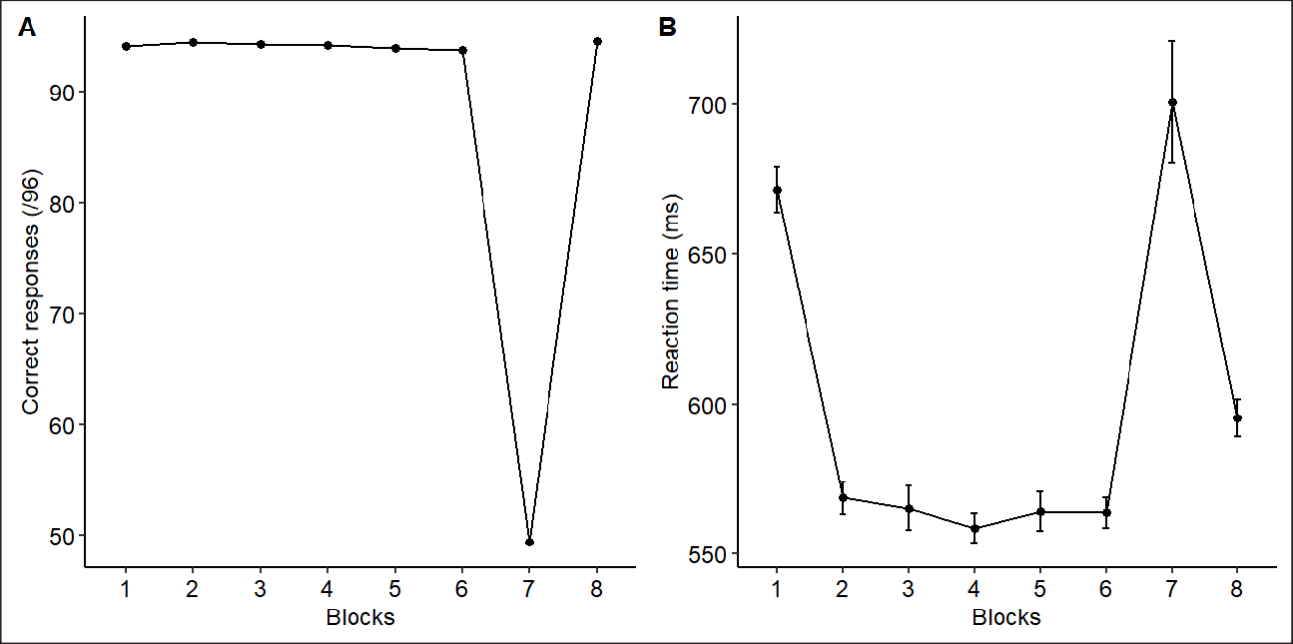
Performance in the SRT task. (A) Correct responses (/96) as a function of blocks (sequential blocks 1 to 6 and 8, random block 7). (B) Mean reaction time (ms) as a function of blocks. Errors bars indicate within-participant standard errors.

Because the RT distribution was slightly skewed, we log-transformed the RTs. This resulted in a more normal RT distribution (Skewness: .74, Kurtosis: 4.24; Blanca et al., 2013). For the LME analysis of the accuracy, the model included by-participant intercepts and random slopes for blocks as random effects. The results showed that the performance of the participants decreased considerably during the 7^th^ random block compared to the other sequential blocks (*b* = –0.47, *SE* = 0.003, *t* = –162.3; see Figure 4A). For the LME analysis of the reaction time of correct responses, the results showed a learning effect during the first 6 blocks, that is, the participants were faster to do the fixed sequence in the 6^th^ sequential block than in the first sequential blocks (*b* = –0.07, *SE* = 0.006, *t* = –12.11). Furthermore, a contrast analysis showed that participants were slower in the 7^th^ random block compared to the sequential blocks just before (*b* = –0.09, *SE* = 0.005, *t* = –20.12) or just after (*b* = 0.07, *SE* = 0.004, *t* = 16.08) (see Figure 4B).

### ASL and VSL tasks

**Figure 5:**
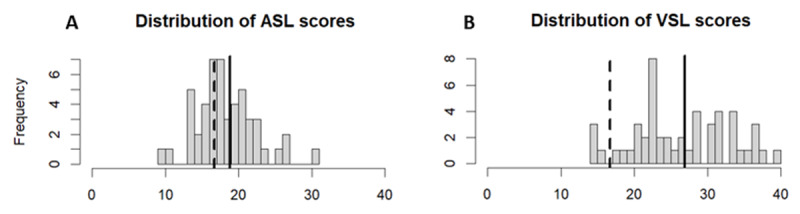
Performance in the auditory (ASL) and visual (CVL) statistical learning task. (A) Distribution of ASL scores. (B) Distribution of VSL scores. The black dashed lines indicate the chance level (16.67 trials) and the solid black lines the group averages.

The average performance on the ASL task was 18.8 trials correct out of 42 trials (SD = 4.13), which was significantly better than the task’s chance-level of 16.67 (*t*(49) = 3.68, p < 0.001) (see Figure 5A). Average performance on the VSL task was 26.9 trials correct out of 42 trials (SD = 6.71), which was significantly better than the task’s chance-level of 16.67 (*t(*49) = 10.80, p < 0.001) (see Figure 5B).

### Correlations

#### Inter-correlations between SL variables

The correlations between the three statistical learning tasks (SRT, ASL, VSL) are presented in Table 1. The analyses showed a significant positive correlation between the ASL and the VSL task (*r* = 0.34, p < 0.05, FDR corrected) but no significant correlations between ASL or VSL and the various SRT measures. Note also that the two SRT measures that take into account performance across repeated blocks (i.e., SRT12, SRT16) correlated with each other. Similarly, significant correlations were also found for the three SRT measures that compared performance in predictive blocks versus performance in random blocks (i.e., SRT67, SRT78, SRT).

**Table 1:** Correlation coefficients for our three statistical learning tasks (SRT, ASL, VSL). Significant Pearson correlations are marked by asterisks: * p < 0.05, ** p < 0.01, *** p < 0.001 (FDR correction).


	VSL^a^	SRT12^b^	SRT16^c^	SRT67^d^	SRT78^e^	SRT^f^

*ASL^a^*	0.336* ***(<.05)***	0.059 *(.801)*	0.074 *(.750)*	–0.149 *(.497)*	–0.144 *(.497)*	–0.168 *(.497)*

*VSL*		–0.127 *(.500)*	–0.165 *(.497)*	–0.136 *(.497)*	0.149 *(.497)*	0.002 *(.987)*

*SRT12*			0.793*** ***(<.001)***	0.102 *(.191)*	–0.015 *(.986)*	0.050 *(.497)*

*SRT16*				0.260*** ***(<.001)***	–0.025 *(.962)*	0.134 *(.083)*

*SRT67*					0.509*** ***(<.001)***	0.877*** ***(<.001)***

*SRT78*						0.859*** ***(<.001)***

*Computed correlation used Pearson-method with listwise-deletion*.						


^a^ Raw score. ^b^ Mean RT of the 1^st^ sequential block – mean RT of the 2^nd^ sequential block. ^c^ Mean RT of the 1^st^ sequential block – mean RT of the 6^th^ sequential block. ^d^ Mean RT of the 7^th^ random block – mean RT of the 6^th^ sequential block. ^e^ Mean RT of the 7^th^ random block – mean RT of the 8^th^ sequential block. ^f^ Mean RT of the 7^th^ random block – mean RT of the 6^th^ and 8^th^ sequential block.

#### Correlations between SL and predictive reading variables

The correlations between the three SL tasks and the predictive reading tasks are presented in Table 2. The analyses showed no significant correlation between semantic or syntactic prediction (or the average of the two) and any of the SL measures. Surprisingly, there was also no significant correlation between semantic and syntactic prediction, which suggests that the two tasks seem to measure different kinds of prediction.

**Table 2:** Correlation coefficients of our three statistical learning tasks (SRT, ASL, VSL) and our predictive reading task. Significant Pearson correlations are marked by asterisks: * p < 0.05, ** p < 0.01, *** p < 0.001 (FDR correction).


	SYNTACTIC PREDICTION	ASL^a^	VSL^a^	SRT12^b^	SRT16^c^	SRT67^d^	SRT78^e^	SRT^f^

*Semantic prediction*	0.065 *(0.83)*	0.088 *(0.83)*	–0.108 *(0.83)*	0.134 *(0.55)*	–0.006 *(0.98)*	–0.077 *(0.75)*	–0.213 *(0.18)*	–0.171 *(0.36)*

*Syntactic prediction*		0.123 *(0.82)*	0.327 *(0.36)*	0.010 *(0.98)*	–0.031 *(0.98)*	0.075 *(0.76)*	–0.153 *(0.41)*	–0.048 *(0.83)*

*Prediction in reading (average)*		0.146 *(0.62)*	0.169 *(0.60)*	0.043 *(0.83)*	–0.022 *(0.91)*	0.034 *(0.85)*	–0.139 *(0.41)*	–0.062 *(0.78)*

*Computed correlation used Pearson-method with listwise-deletion*.								


^a^ Raw score. ^b^ Mean RT of the 1^st^ sequential block – mean RT of the 2^nd^ sequential block. ^c^ Mean RT of the 1^st^ sequential block – mean RT of the 6^th^ sequential block. ^d^ Mean RT of the 7^th^ random block – mean RT of the 6^th^ sequential block. ^e^ Mean RT of the 7^th^ random block – mean RT of the 8^th^ sequential block. ^f^ Mean RT of the 7^th^ random block – mean RT of the 6^th^ and 8^th^ sequential block.

#### Correlations between reading level, SL, and predictive reading variables

The correlations between the predictive reading scores and the reading level and the reading speed of our participant are presented in Table 3. The analyses showed a significant negative correlation between overall reading level in the standardized reading task and overall reading speed in the predictive reading task (r = –0.33, p < 0.001, FDR corrected). A negative correlation means that people with a good reading/comprehension ability are also faster readers in our experimental task. Interestingly, we found a significant positive correlation between our predictive reading score and overall reading ability (r = 0.21, p < 0.05, FDR corrected). This correlation indicates that good readers/comprehenders tend to be good language predictors. Given that reading ability and reading speed are correlated negatively, it is not surprising to also find a significant negative correlation between the participants’ reading speed in our experimental task and their predictive reading score (r = –0.20, p < 0.05, FDR corrected). This negative correlation suggests that faster readers are better language predictors or the other way around.

**Table 3:** Correlation coefficients between the reading level and the reading speed of our participant, and the predictive reading measures. Significant Pearson correlations are marked by asterisks: * p < 0.05, ** p < 0.01, *** p < 0.001 (FDR corrected).


	READING SPEED	PREDICTION IN READING	SEMANTIC PREDICTION	SYNTACTIC PREDICTION

*Reading level*	–0.327*** ***(<.001)***	0.207* ***(<.05)***	0.101 *(.369)*	0.208 *(.070)*

*Reading speed*		–0.204* ***(<.05)***	–0.195 *(.085)*	–0.178 *(.104)*

*Computed correlation used Pearson-method with listwise-deletion*.				


The correlations between the three statistical learning tasks (SRT, ASL, VSL) and reading level and reading speed are presented in Table 4. The analyses showed a significant positive correlation between overall reading ability and the ASL score (r = 0.42, p < 0.01, FDR corrected). Reading ability also correlated with SRT67, one of the SRT measures that is based on the drop of performance in the random block that follows a predcitive block (r = 0.24, p < 0.05, FDR corrected). These correlations suggest that good readers/comprehenders tend to be good statistical learners in the auditory modality and to some extent in the motor modality. We also found a significant negative correlation between the participants’ reading speed in our predictive reading task and the SRT67 score (r = –0.28, p < 0.05, FDR corrected), which indicates that fasters readers tend to be better implicit learners. These correlations could also be interpreted the other way around: good statistical learners and good language predictors tend to be better readers/comprehenders.

**Table 4:** Correlation coefficients between the reading level and the reading speed of our participant, and our three statistical learning tasks (SRT, ASL, VSL). Significant Pearson correlations are marked by asterisks: * p < 0.05, ** p < 0.01, *** p < 0.001 (FDR corrected).


	ASL^a^	VSL^a^	SRT12^b^	SRT16^c^	SRT67^d^	SRT78^e^	SRT^f^

*Reading level*	0.419** ***(<.01)***	0.282 *(.142)*	0.107 *(.455)*	0.110 *(.455)*	0.236* ***(<.05)***	0.023 *(.929)*	0.149 *(.250)*

*Reading speed*	–0.211 *(.319)*	0.048 *(.891)*	–0.094 *(.475)*	0.010 *(.986)*	–0.276* ***(<.05)***	0.005 *(.986)*	–0.156 *(.231)*

*Computed correlation used pearson-method with listwise-deletion*.							


^a^ Raw score. ^b^ Mean RT of the 1^st^ sequential block – mean RT of the 2^nd^ sequential block. ^c^ Mean RT of the 1^st^ sequential block – mean RT of the 6^th^ sequential block. ^d^ Mean RT of the 7^th^ random block – mean RT of the 6^th^ sequential block. ^e^ Mean RT of the 7^th^ random block – mean RT of the 8^th^ sequential block. ^f^ Mean RT of the 7^th^ random block – mean RT of the 6^th^ and 8^th^ sequential block.

## Discussion

There is a growing interest in the potential link between SL and normal and impaired reading. Some studies have found that SL is associated with reading (Arciuli & Simpson, 2012) and sentence processing (Misyak & Christiansen, 2012), while others have found null correlations with reading or reading-related skills (Haebig et al., 2017; Schmalz et al., 2019; Siegelman & Frost, 2015).

Because all previous studies investigated quite general levels of reading ability (e.g., Arciuli & Simpson, 2011; Misyak & Christiansen, 2012), in the present study, we used a predictive reading task, in which participants could use semantic or syntactic contexts to facilitate the processing of a target word (Gavard & Ziegler, 2022). This task results in semantic and syntactic context effects that allowed us to calculate for each participant a linguistic prediction score (i.e., a semantic or syntactic priming effect). We hypothesized that, if anything, SL measures should be more strongly related to the ability to make linguistic predictions rather than global measures of reading speed or reading ability.

We will now summarize and discuss our findings in the context of the extant literature on SL and reading. First of all, we replicated the semantic and syntactic context effects on reading aloud (Gavard & Ziegler, 2022) in an on-line experiment using a large sample of participants (N = 120). Incidentally, this finding nicely demonstrates that reading aloud experiments can be conducted online and produce high-quality results (Fairs & Strijkers, 2021). We also replicated the finding that syntactic context effects were stronger than semantic context effects despite the fact syntactic contexts cannot activate precise lexical entries, whereas semantic contexts could possibly pre-activate specific lexical entries. Our results support the findings of Snell and Grainger (2017), who found faster partial report word identification accuracy for words that were embedded in syntactically correct sequences as opposed to scrambled agrammatical sequences. Taken together, these results suggest that syntactic contexts provide a strong constraint on single word identification and reading aloud. It is also noteworthy that there was no correlation between semantic and syntactic prediction scores, which means that these two types of linguistic predictions are quite specific and do not seem to depend on a single common mechanism. It is possible that semantic priming is more associated with lexical processing and attention (i.e., pay attention to the context and activate lexical candidates that fit the semantic category), whereas syntactic priming might reflect more general language skills and working memory (i.e., activate a sentence frame in short term memory and predict the syntactic role of the target word).

Second, we did not find the expected link between semantic and/or syntactic prediction effects and SL abilities. One explanation might be that our predictive reading task was not suited for investigating interindividual differences although it produced quite strong effects at the group level. To address this concern, we assessed the test-retest reliability of this task and we found a reasonable test-rest reliability of *r* = .56 for our prediction scores. As a comparison, Perry, Ziegler and Zorzi (2010) looked at the variance that is reproducible for the same items across different reading aloud databases and found that performance to identical items across four different databases correlated moderately, with *r* values ranging between .42 and .68 (i.e., 17.6% and 46.2% of the variance). Thus, the test-retest reliability of our prediction scores is quite close to the amount of variance that is reproducible for the same items in reading aloud (see also Perry, Evertz, Zorzi, & Ziegler, 2024). Together then, our results are not in favor of the idea that the ability to make linguistic predictions is based on a domain-general SL ability.

Third, although we failed to find a link between SL and linguistic prediction, there was a significant link between SL in the auditory segmentation task (ASL) and general reading ability, as measured by reading aloud speed in the predictive reading task and reading ability in the standardized reading test. The size of the correlation (*r* = 0.419) was comparable to that recently reported by Ren et al. (2023) in a meta-analysis on SL and reading (*r* = 0.260). Thus, it seems to be the case that SL is related more generally to reading proficiency than more specifically to linguistic prediction. This could be because SL might have a very strong effect during reading development (Seidenberg & MacDonald, 2018), which in turn affects general reading proficiency. In contrast, linguistic predictions might rely on higher level factors, such as memory, attention, or vocabulary. In support of this idea, Mani and Huettig (2012) showed that children’s linguistic prediction skills were significantly correlated with their productive vocabulary size (i.e., children with large production vocabularies were better at predicting upcoming linguistic input).

Fourth, we also replicated the finding that auditory SL seems to show stronger correlations with general reading ability than visual SL (Gabay et al., 2015). This result is in line with a previous study which showed that reading skills were more strongly associated with auditory SL than with visual SL in typically reading adults and children (Qi et al., 2019). It is also consistent with a recent study on dyslexia which showed that adults with dyslexia exhibited typical statistical learning for visual material, but impaired statistical learning for auditory material and only auditory statistical learning correlated positively with single-word reading performance both across all participants and in the group of dyslexics (Ozernov-Palchik et al., 2023).

Fifth, the two types of SL tasks were not correlated suggesting that there was a clear dissociation between the two classes of SL tasks (SRT vs. ASL/VSL). As for the predictive reading tasks, this could be possibly due to the fact that, unlike the ASL and VSL tasks, the SRT task was not optimized for the investigation of interindividual differences. However, this explanation does nots seem to hold because the test-retest reliability of the SRT task was actually quite high (r = .62). Thus, it is more plausible that these tasks measure different aspects of SL. Indeed, SRT is a spatial-motor skill learning task. Skill learning is a form of implicit learning that involves the acquisition of perceptual-motor skills through practice and feedback and relies on the procedural memory system (Nicolson & Fawcett, 2007, 2011; Ullman, 2004). In that respect, VSL and ASL segmentation tasks are quite different because they do not require explicit knowledge of the task goal or any motor engagement (Batterink et al., 2015; Song et al., 2007). The findings in the dyslexia study by Ozernov-Palchik et al. (2023) are clearly in favor of a dissociation between these two types of SL tasks because they found deficits in adults with dyslexia in an ASL segmentation task but not in skill learning on mirror tracing and rotary pursuit tasks, two tasks that reflect purely procedural memory.

The differences between SL tasks in relation to reading and dyslexia are clearly consistent with the pluralist view of SL (Frost et al., 2015), according to which SL across modalities and domains operates through partially overlapping, but yet distinct mechanisms. If we take the componential view of SL, as advocated by Arciuli and colleagues (2012, 2018), it seems clear that extracting and learning auditory patterns from the environment (ASL) is more relevant to reading than implicit skill learning. In this respect, it is also less surprising why we did not find a link to the ability to make linguistic predictions because the nature of these tasks and the possibly associated SL computations are rather different. Performance in SL is a snapshot of a participant’s ability to learn about sensory regularities. In contrast, the ability to make linguistic predictions is the end product of a long-lasting learning process that might be influenced by many other factors. Without a precise theory about the kinds of computations involved in different domains and modalities, it is unlikely to gain a better understanding of the link between SL and linguistic predictions skills.

## Data Accessibility Statement

The information needed to reproduce all the reported results and methodology is available at https://osf.io/e2msk/.

## Footnotes

www.prolific.co.

The 16 sounds are available online at: htpp://osf.io/x25tu.

www.labvanced.com.

## Appendix

**Table S1:** Psychometric properties of all variables with their distributions

VARIABLES	N	MIN	MAX	MEAN	ST.DEV	SKEWNESS	KURTOSIS	HISTOGRAM

Reading level	120	25	77	56.98	13.51	–0.60	–0.53	
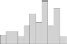

Reading speed	120	436.7	974	660.4	111.98	0.60	–0.17	
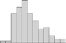

Prediction in reading (average)	120	–0.04	0.12	0.03	0.03	0. 75	2.63	
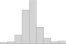

Semantic prediction	94	–0.05	0.09	0.02	0.03	0.38	1.02	
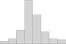

Syntactic prediction	96	–0.06	0.12	0.04	0.03	0.29	0.60	
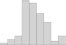

ASL	50	9	31	18.82	4.13	0.45	0.74	
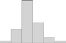

VSL	50	14	40	26.92	6.71	–0.008	–0.85	
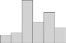

SRT	120	–0.03	0.68	0.20	0.11	0.92	2.49	
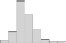

SRT12	120	–0.08	0.72	0.16	0.11	1.65	5.81	
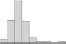

SRT16	120	–0.09	0.97	0.16	0.15	1.94	7.70	
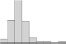

SRT78	120	–0.19	0.58	0.17	0.12	0.27	1.34	
